# PD-L1 Expression Predicts a Distinct Prognosis in Krukenberg Tumor with Corresponding Origins

**DOI:** 10.1155/2018/9485285

**Published:** 2018-05-08

**Authors:** Haiyan Tai, Qin Yang, Zhiyong Wu, Su'an Sun, Rui Cao, Yanfeng Xi, Ran Zhao, Mengyu Zhang, Zhigang Zhang, Congjian Xu

**Affiliations:** ^1^Obstetrics and Gynecology Hospital, Fudan University, Shanghai, China; ^2^State Key Laboratory of Oncogenes and Related Genes, Shanghai Cancer Institute, Shanghai Jiao Tong University, Shanghai, China; ^3^Department of Pathology, The First Hospital of Huai'an City, Nanjing Medical University, Huai'an, Jiangsu Province, China; ^4^Dalian Obstetrics and Gynecology Hospital, Dalian, Liaoning Province, China; ^5^Department of Pathology, Shanxi Provincial Cancer Hospital, Shanxi Medical University, Taiyuan, Shanxi, China; ^6^Department of Gynecology, 411 Military Hospital, Shanghai, China; ^7^Department of Obstetrics and Gynecology of Shanghai Medical School, Fudan University, Shanghai, China; ^8^Shanghai Key Laboratory of Female Reproductive Endocrine Related Diseases, Shanghai, China

## Abstract

Krukenberg tumor (KT) is an uncommon ovarian metastatic signet-ring cell adenocarcinoma that mostly metastasizes from gastrointestinal carcinoma. Optimal treatment options for KTs are limited. Programmed death-1 (PD-1)/programmed death-ligand 1 (PD-L1) inhibitors have shown remarkable activity in clinical trials for metastatic tumors. Here, we evaluated PD-L1 expression and T cell infiltration in KTs and their corresponding primary tumors. Positive tumor PD-L1 expression was detected in 9 (25.7%) KTs from gastric carcinomas (GCs) and in 20 (66.7%) KTs from colorectal carcinomas (CRCs). Patient survival was assessed according to the PD-L1 status and CD8^+^ T cell density. Positive tumor PD-L1 expression in KTs from GCs was associated with poor prognosis. In contrast, positive tumor PD-L1 expression in KTs from CRCs was associated with an improved prognosis. We analyzed copy number variations of the *PD-L1* gene in KTs. PD-L1 expression was higher in cases with copy number gains. The T cell densities within KTs and their corresponding primary tumors were compared. The densities of CD8^+^ T cells correlated significantly between the primary tumors and KTs from the same case. Taken together, the research further highlighted targets for immune-based therapy in KTs from GCs and CRCs.

## 1. Introduction

Ovarian metastatic tumors that contain a component of signet-ring cells are known as Krukenberg tumors (KTs) and originate mainly from the stomach (76%), intestines (11%), breast (4%), and other organs [1]. KTs chiefly affect premenopausal women. Although a few studies have suggested that patients might benefit from metastasectomy with systemic chemotherapy, optimal treatment options are limited and the prognosis is poor [2–4]. Most patients die within 2 years (median survival time: 14 months) [5]. Thus, the need for new strategies to treat KTs is pressing.

Immunotherapy has emerged as a promising measure for cancer treatment [6]. Accumulative data have revealed the successful application of immune checkpoint blockers in multiple cancer types, including advanced gastrointestinal carcinoma [7–10]. In one clinical trial, high response rates to PD-1 inhibitors were observed among advanced colorectal carcinoma (CRC) and gastric carcinoma (GC) patients whose tumors were mismatch repair-deficient [10]. The relationship between PD-L1/PD-1 expression and therapeutic response was evident, and none of the PD-L1-negative tumors responded [11]. Thus, a study of tumor PD-L1 expression in ovarian metastases from gastrointestinal cancer is needed.

We investigated PD-L1 expression in KTs from GCs and CRCs, examined the correlation between PD-L1 expression and T cell infiltration, and evaluated the impact of PD-L1 expression on prognosis. Copy number variations in the *PD-L1* gene in KTs from GCs were analyzed. The immune microenvironment of KTs was also assessed and compared with that of the primary tumor from the same case.

## 2. Materials and Methods

### 2.1. Case Cohort

We reviewed a retrospective cohort study of 65 cases. A tissue microarray was constructed for paraffin sample tissues and included 35 KTs with 23 matched primary GCs and 30 KTs with 28 matched primary CRCs collected at Fudan University Affiliated Obstetrics and Gynecology Hospital and cooperative hospitals between 2000 and 2015. The overall survival (OS) time was defined as the interval between the ovarian metastasectomy operation and death or survival. Exclusion criteria included (a) the absence of surgery of KT and (b) the validation of an ovarian nonadenocarcinoma metastasis. Samples and medical records were approved by the research ethics committee of Fudan University Affiliated Obstetrics and Gynecology Hospital and cooperative hospitals.

### 2.2. Immunohistochemistry

The primary antibodies used were as follows: anti-PD-L1 for immunohistochemistry (IHC, rabbit monoclonal antibody, Abcam, UK, ab205921, 1 : 100), anti-PD-L1 for multiplex IHC (rabbit monoclonal antibody, CST, USA, 78701, 1 : 200), anti-PD-1 (mouse monoclonal antibody, CST, USA, 43248, 1 : 100), anti-FOXP3 (rabbit monoclonal antibody, CST, USA, 98377, 1 : 50), anti-CD3 (rabbit monoclonal antibody, Abcam, UK, ab16669, 1 : 100), anti-CD8 (mouse monoclonal antibody, Abcam, UK, ab11147, 1 : 25), and anti-CD8 for multiplex IHC (mouse monoclonal antibody, CST, USA, 78701, 1 : 250). Positive staining was visualized with DAB substrate liquid (CST, USA), and counterstaining was performed with hematoxylin. Scoring was performed by two senior pathologists.

Tumor PD-L1 expression was determined by the presence of membrane staining in tumor cells as previously reported [12]. Stromal PD-L1 expression was evaluated according to the presence of membrane staining in stromal cells. The PD-1^+^, FOXP3^+^, CD3^+^, and CD8^+^ cell densities (cells/mm^2^) were quantified using digital image analysis. The CD8^+^ T cell densities were dichotomized into “high” and “low” groups according to the median.

### 2.3. Multiplex Immunochemistry

The tissue sections were deparaffinized, rehydrated, and incubated with 0.3% hydrogen peroxide, and the antigen was unmasked in 10 mM sodium citrate buffer using a microwave. After the sections were incubated with the primary antibody for 45 min at RT, slides were incubated with anti-rabbit secondary antibody (NEF812E001EA; PerkinElmer) or anti-mouse secondary antibody (NEF822E001EA; PerkinElmer) for 30 min at RT. Tyramide (TSA) plus fluorescein (NEL741E001KT, PerkinElmer) or TSA plus Cyanine 5 (NEL745E001KT, PerkinElmer) was added to the slides at a 1 : 50 dilution, and the slides were incubated for 10 min at RT. A microwave was used to remove the combined antibody. TSA plus fluorescein or TSA plus Cyanine 5 was added to slides at a 1 : 50 dilution. Cell nuclei were counterstained with DAPI. The slides were imaged using Vectra imaging software.

### 2.4. Copy Number Analysis

The *PD-L1* copy number in 13 paraffin samples was analyzed using the OncoScan formalin-fixed and paraffin-embedded (FFPE) Assay Kit (Affymetrix; USA). Total FFPE DNA was extracted using the QIAamp DNA Mini Kit (Qiagen; Germany). Results were analyzed using ChAS (Chromosome Analysis Suite, version 3.1.1.27) software. Raw data was submitted to ArrayExpress, and the data accession number is E-MTAB-6277.

### 2.5. Statistical Analysis

Statistical analyses were conducted using Statistical Product and Service Solutions 16.0 software (Chicago, IL, USA). Chi-square tests in cross tables were performed to examine the correlation between the PD-L1 expression levels and clinical parameters or lymphocyte variables. Correlations of T cell infiltration between the primary tumor and metastases were calculated with the Spearman correlation test. OS was estimated using the Kaplan-Meier method, and differences were evaluated using the log-rank test. Graphic representations were analyzed with GraphPad Prism software (San Diego, CA). *P* values < 0.05 were considered to be statistically significant.

## 3. Results

### 3.1. Clinicopathological Findings

A retrospective cohort study of 65 patients, including 35 KTs from GCs and CRCs, was conducted. The clinicopathological characteristics of the patients are detailed (Supplementary Tables S1 and S2). The median age of the patients with KTs from GCs was 41 (22–62) years. The median OS time was 9 months (9 ± 1.7 months). Among the 35 cases, tumors were more common in premenopausal women (82.9%) than in postmenopausal women (17.1%). Most patients (82.9%) had synchronous ovarian metastasis. The median age of the patients with KTs from CRCs was 50 (30–79) years. The median OS time was 17 months (17 ± 2.397). Among the 30 cases, postmenopausal women (53.3%) and premenopausal women (46.7%) were equally represented.

### 3.2. Expression of PD-L1 in KTs and Its Association with Clinicopathological Parameters

**Figure 1:**
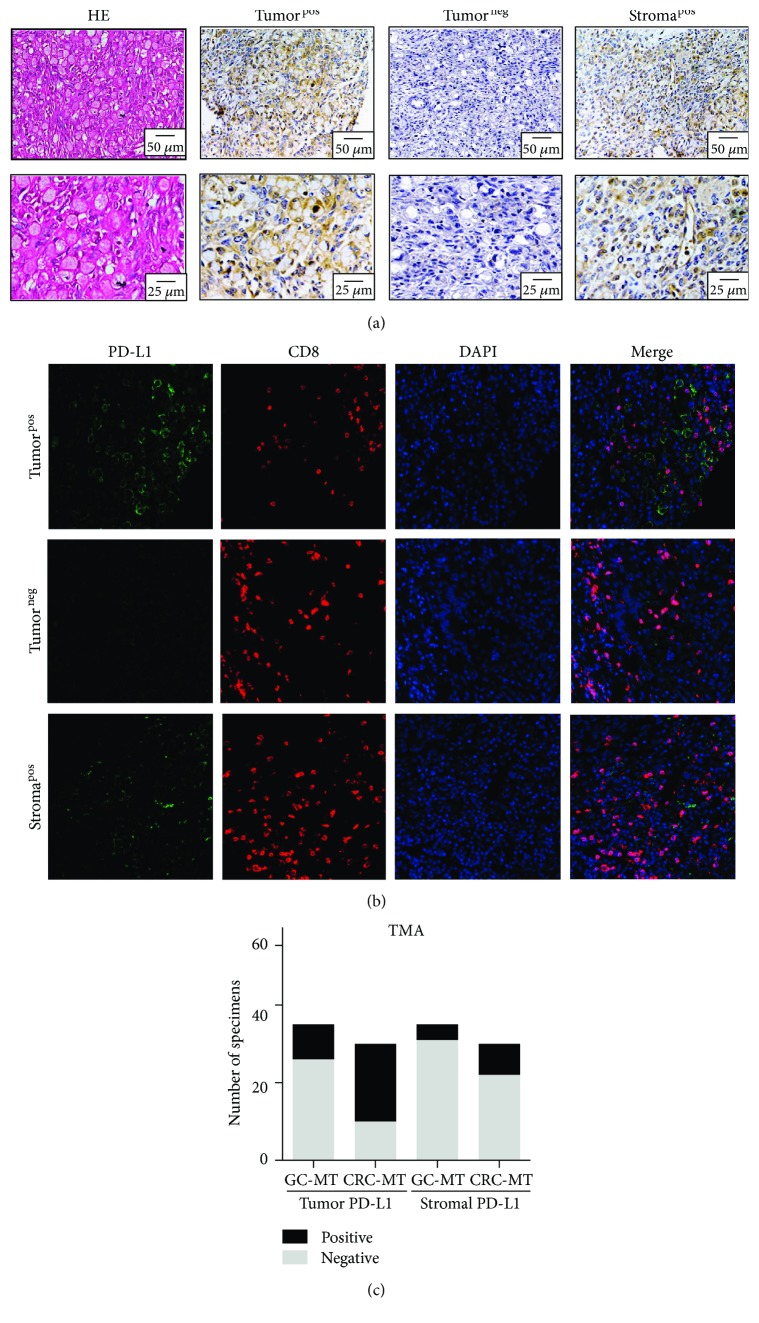
PD-L1 expression in KTs. (a) Representative samples of HE and IHC staining of PD-L1 were shown, including tumor cells, stromal cells, and PD-L1-negative tumors. (b) Multiplex IHC of PD-L1 and CD8 on tumor cells and stromal cells. Nuclei were stained with DAPI. (c) The percentage of the tissue displaying positive and negative tumor and immune stromal PD-L1 staining in KTs from the GCs and CRCs. MT: metastasis.

PD-L1 was expressed on the cell membranes of the tumor cells or stromal cells (Figure 1(a)). Tumor PD-L1 expression was detected in 9 (25.7%) KTs from GCs and 20 (66.7%) KTs from CRCs. Stromal PD-L1 expression was detected in 4 (11.4%) KTs from GCs and 8 (26.7%) KTs from CRCs (Figure 1(c)). The expression of the tumor and stromal PD-L1s was evaluated by multiplex IHC techniques (Figure 1(b)).

The relationship between the tumor or stromal PD-L1 expression and the clinical characteristics of the patients with ovarian metastasis are shown (Supplementary Tables S1 and S2). In the GC patients, we observed that the tumor PD-L1 expression was positively associated with postmenopausal status (*P* = 0.027). In the CRC patients, an inverse relationship between the tumor PD-L1 expression and the extent of signet-ring cells was observed (*P* = 0.009). No significant relationship was evident between the stromal PD-L1 expression and any clinicopathological feature (Supplementary Tables S1 and S2).

### 3.3. PD-L1 Expression and CD8^+^ T Cell Infiltration Are Associated with Overall Survival

**Figure 2:**
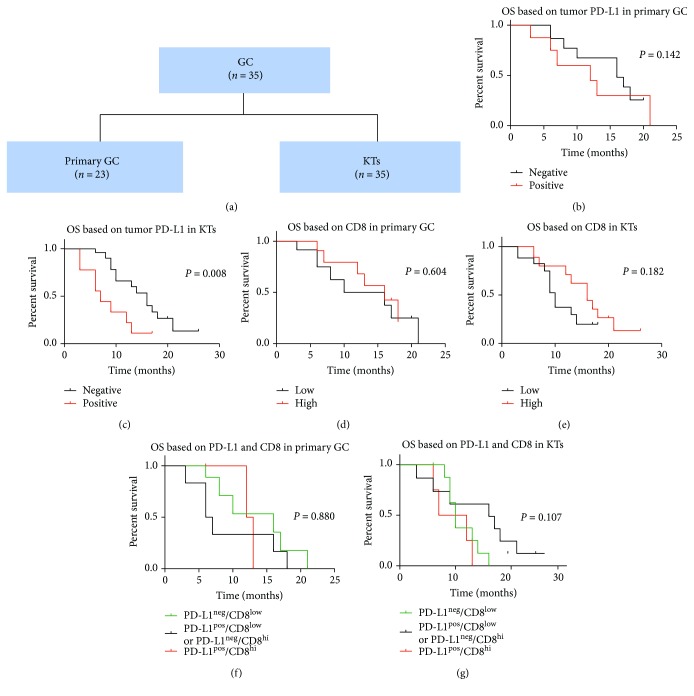
Prognostic value of tumor PD-L1 expression and CD8 T cell densities in the primary GCs and KTs. Sample cohorts (a). Kaplan-Meier survival curves for OS according to the PD-L1 expression in primary GCs (b) and KTs (c). Kaplan-Meier survival curves for OS according to the CD8 T cell densities in primary GCs (d) and KTs (e). Kaplan-Meier survival curves for OS according to a combined analysis of the PD-L1 expression and CD8 T cell densities in primary GCs (f) and KTs (g).

**Figure 3:**
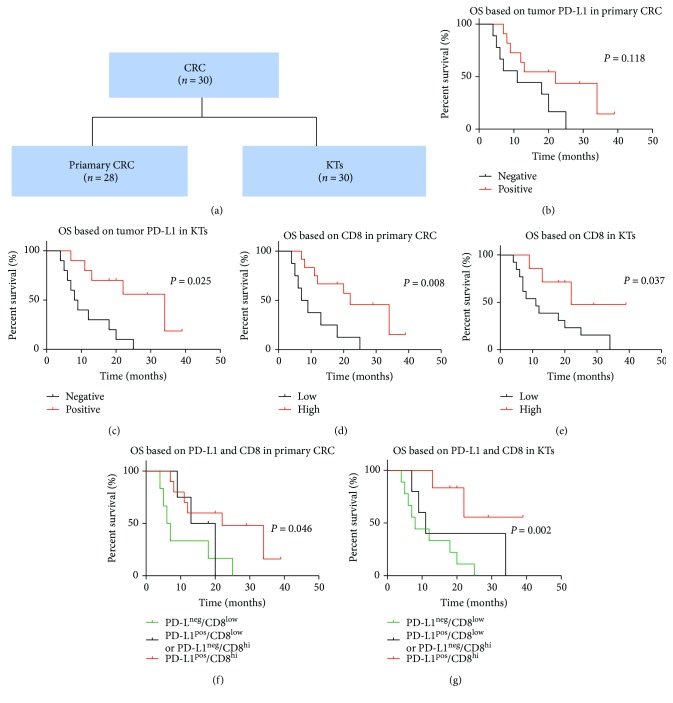
Prognostic value of tumor PD-L1 expression and CD8 T cell densities in the primary CRCs and KTs. Sample cohorts (a). Kaplan-Meier survival curves for OS according to the PD-L1 expression in primary CRCs (b) and KTs (c). Kaplan-Meier survival curves for OS according to the CD8 T cell densities in primary CRCs (d) and KTs (e). Kaplan-Meier survival curves for OS according to a combined analysis of the PD-L1 expression and CD8 T cell densities in primary CRCs (f) and KTs (g).

Kaplan-Meier analysis was performed to evaluate OS according to the tumor or stromal PD-L1 expression in KTs (Figures 2 and 3). Positive tumor PD-L1 expression in KTs from GCs was associated with worse OS than negative tumor PD-L1 expression (*P* = 0.008, Figure 2(c)). Conversely, positive tumor PD-L1 expression in KTs from CRCs was associated with an improved OS compared to negative tumor PD-L1 expression (*P* = 0.025, Figure 3(c)).

The impact of tumor PD-L1 expression in primary tumors on OS was also determined. Positive tumor PD-L1 expression had a tendency for poor prognosis in the GC cases (*P* = 0.142, Figure 2(b)) and for an improved prognosis in the CRC cases compared to negative tumor PD-L1 expression (*P* = 0.118, Figure 3(b)).

Because PD-L1/PD-1 signaling might be secondary to CD8^+^ T cell infiltration [13], we quantified CD8^+^ T cell infiltration in the ovarian metastases. However, no significant correlation was evident between the tumor or stromal PD-L1 expression and the CD8^+^ density (Supplementary Tables S3 and S4). Kaplan-Meier analysis was performed to evaluate OS according to the density of CD8^+^ T cells in the ovarian metastases. High densities of infiltrating CD8^+^ T cells were associated with prolonged OS in the GC ovarian metastases (*P* = 0.182, Figure 2(e)) and in the CRC ovarian metastases (*P* = 0.037, Figure 3(e)). For the CRC patients, the combination of the positive tumor PD-L1 expression and a high CD8^+^ T cell density was associated with a better OS rate than either factor alone (*P* = 0.002, Figure 3(g)).

Characteristics including PD-L1 expression in ovarian metastases were analyzed using COX proportion hazard regression models to assess the prognostic values (Table 1). In GC patients, the positive tumor PD-L1 expression (HR = 3.201 (95% CI: 1.273–8.050), *P* = 0.013) and menopause status (HR = 2.860 (95% CI: 1.057–7.736), *P* = 0.038) were analyzed to be risk factors for OS.

In CRC patients, the negative tumor PD-L1 expression in metastases (HR = 5.129 (95% CI: 1.567–16.791), *P* = 0.007) and high ratio of signet cells (HR = 4.655 (95% CI: 1.366–15.863), *P* = 0.014) were identified as potential prognostic factors (Table 2). The negative tumor PD-L1 expression retained its significance in the multivariate analysis.

### 3.4. Copy Number Variation of PD-L1 and Its Association with PD-L1 Expression

**Figure 4:**
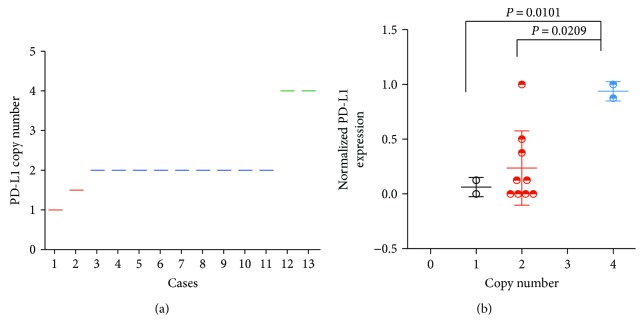
*PD-L1* copy number status and PD-L1 expression in KTs. (a) *PD-L1* copy number status in 13 KTs. (b) A higher PD-L1 expression was observed in cases with copy number gains than in normal cases (*P* = 0.0209) or cases with copy number losses (*P* = 0.0101).

Aiming to evaluate the impact of copy number alterations on PD-L1 expression, we performed a copy number analysis of 13 KTs from GCs with the OncoScan FFPE assay adjusted to 13 normal control tissues. The copy number in each case was analyzed, and the results revealed *PD-L1* gene copy number gains in 2 cases (15.4%), *PD-L1* gene copy number losses in 2 cases (15.4%), and a normal copy number status in 9 cases (69.2%). The PD-L1 expression was higher in cases with copy number gains than in normal cases (*P* = 0.0209) or cases with copy number losses (*P* = 0.0101) (Figure 4).

### 3.5. Association of PD-L1 Status with T Cell Infiltration

Although DNA copy number gains play an important role in gene overexpression, the evidence explaining the deregulation of PD-L1 was insufficient. The immunological microenvironment is reportedly involved in tumor PD-L1 upregulation [13]. The associations between tumor PD-L1 expression and T cell densities in KTs were analyzed. The tumor PD-L1 expression score was not significantly associated with CD3^+^, CD8^+^, FOXP3^+^, or PD-1^+^ T cell infiltration (Supplementary Tables S3 and S4).

### 3.6. T Cell Infiltration in Primary Tumor and KTs

**Figure 5:**
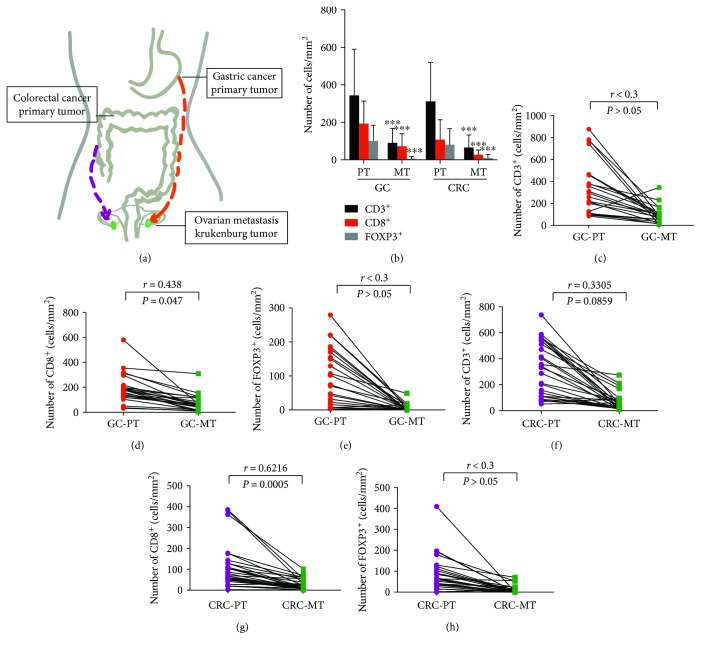
CD3^+^, CD8^+^, and FOXP3^+^ cell densities in the primary tumor and KTs. (a) Schematic diagram of the metastasis process. (b) Primary GCs and CRCs are more infiltrated by CD3^+^, CD8^+^, and FOXP3^+^ T cells than the KTs. (c–e) The *R-*value shows the correlations between the primary GCs and KTs according to CD3^+^, CD8^+^, and FOXP3^+^ T cell densities. (f–h) The *R*-value shows the correlations between the primary CRCs and KTs according to CD3^+^, CD8^+^, and FOXP3^+^ T cell densities. ^∗∗∗^ *P* < 0.001; PT: primary tumor; MT: metastasis.

We compared T cell densities in the primary GCs to those in the KTs from the same individuals (*n* = 23). The densities of CD8^+^, CD3^+^, and FOXP3^+^ cells were significantly higher in the primary GCs than in the KTs (*P* < 0.001; Figure 5(b)). Furthermore, the densities of CD8^+^ cells were positively correlated between the primary tumors and KTs (*r* = 0.438, *P* = 0.047, Figure 5(d)). However, the densities of CD3^+^ and FOXP3^+^ cells were not correlated between the primary tumors and KTs (*r* < 0.3, Figures 5(c) and 5(e)).

We also compared T cell densities in the primary CRCs to those in the KTs (*n* = 28). Similarly, the densities of CD8^+^, CD3^+^, and FOXP3^+^ cells were significantly higher in the primary CRCs than in the KTs (*P* < 0.001; Figure 5(b)). CD8^+^ cells were positively correlated between the primary tumors and KTs (*r* = 0.6216, *P* = 0.0005; Figure 5(g)). CD3^+^ cells and FOXP3^+^ cells were not correlated between the primary tumors and KTs (Figures 5(f) and 5(h)).

## 4. Discussion

Given the success of PD-L1/PD-1 inhibitors in metastatic tumors, we conducted this study to characterize the role of PD-L1 and the immune microenvironment in KTs. In KTs from GCs, positive tumor PD-L1 expression was associated with poor prognosis. In contrast, in KTs from CRCs, positive tumor PD-L1 expression was associated with an improved prognosis compared to negative PD-L1 expression. The GCs and CRCs had patterns of CD8^+^ cells that were correlated between the primary tumors and KTs from the same case.

Although metastatic spread is the main cause of cancer-related death, patients with metastases have heterogeneous survival outcomes. For KTs, survival is associated with the primary tumor [14, 15]. In our study, the CRC patients showed a longer OS time (17 ± 2.397 months) than the GC patients (9 ± 1.7 months), which was consistent with previous studies [14, 15]. To address the prognostic value of tumor PD-L1, we performed a Kaplan-Meier survival analysis and found that the tumor PD-L1 expression in KTs with a distinct tumor origin was associated with a different prognosis. Previous studies have demonstrated an association between high PD-L1 expression and poor prognosis in primary GC [12, 16], which was consistent with our study. High PD-L1 expression is reportedly associated with a better prognosis than low PD-L1 expression in several tumor types [17–19], including primary CRC; however, conflicting results have been reported for CRC [17, 20].

Programmed death-ligand 1 (PD-L1) expression is upregulated in multiple human cancers and attenuates the antitumor immune response [21–24]. Two mechanisms for the upregulation of PD-L1, including adaptive resistance and intrinsic immune resistance, have been proposed. Adaptive resistance occurs when PD-L1/PD-1 serves as a negative feedback mechanism that follows CD8^+^ T cell infiltration and is driven by the immune system [13, 23, 25]. Intrinsic resistance leads to PD-L1 upregulation due to a copy number gain, mutations, or oncogenic signaling within the tumor [26].

Genome abnormalities might affect the tumor PD-L1 expression [10]. A previous study had reported that the PD-L1 expression was significantly higher in cases with PD-L1 copy number gain than in normal cases in thymic carcinoma [27]. Consistent with this observation, our study showed that tumor cell PD-L1 expression was higher in cases with copy number gains. In addition to copy number aberrations, oncogenic mutations might also participate in the mechanism of PD-L1 overexpression [10]. Thus, further studies on genetic aberrations are essential to improve treatment methods.

Although DNA copy number gains play important roles in gene overexpression, *PD-L1* gene amplification was identified in only 2 of 13 samples, and this evidence was insufficient to explain the overexpression of PD-L1. Previous studies have reported a significant relationship between tumor PD-L1 expression and the CD8^+^ cell density [12, 28]. IDO, PD-L1, and FOXP3^+^ regulatory T cell inhibitory pathways might serve as a negative feedback mechanism that follows CD8^+^ T cell infiltration [13]. Another study has shown a significant inverse association between the tumor PD-L1 expression and FOXP3^+^ cell density [29]. However, we observed no significant association between the tumor PD-L1 expression or stromal PD-L1 expression and CD3^+^, CD8^+^, or FOXP3^+^ T cell density, which may be due to differences in the study populations.

We further evaluated the immune contexture of the primary tumors and KTs. From the primary tumors to the metastases, the GCs and CRCs had significant correlation patterns between CD8^+^ cells. Consistently, previous studies have demonstrated that the immune contexture in the primary tumor results in “educated” immune cells that are recalled at the metastatic sites [30]. Tumor cells reportedly imprint their microenvironments during all disease stages, with a similar architecture and clinical impact [30–32]. Our study implies that the immune contexture may be partially reproducible from the primary tumor to metastatic tumors and may affect tumor PD-L1 expression.

Our study has some limitations. First, most of the KTs were over 5 cm in size and most were associated with no normal tissues in the margins. Thus, we were not able to assess the tumor and stromal PD-L1 expressions and the immune cell infiltration within the tumor-invasive margin. Second, the study included a small sample size because (i) Krukenberg tumor is uncommon, only accounting for 1% to 2% of all ovarian tumors [5]; (ii) whether an ovarian surgical resection should be performed has not been established. Samples are difficult to obtain. However, more samples are needed to confirm our hypothesis.

In conclusion, positive tumor PD-L1 expression implies that there might be implications for targeting the PD-L1/PD-1 axis in KTs. The prognostic value and immune pattern in the metastases might be useful for guiding treatment.

## 5. Conclusions

Our results characterized the role of PD-L1 and the immune microenvironment in KTs. Tumor PD-L1 expression predicted diverse prognoses in KTs with different corresponding origins. In KTs from GCs, positive tumor PD-L1 expression was associated with poor prognosis. In contrast, in KTs from CRCs, the positive tumor PD-L1 expression was associated with an improved prognosis compared to the negative PD-L1 expression. The GCs and CRCs had patterns of CD8^+^ cells that were correlated between the primary tumors and KTs from the same case, indicating that the primary tumor exerted an influence on the immune environment in the metastases. It further highlighted targets for immune-based therapy in KTs from GCs and CRCs.

## Figures and Tables

**Table 1:** Univariate and multivariate analysis for prognostic factors affecting OS in GC.

Risk factors	Univariate analysis		Risk factors	Multivariate analysis	
	HR (95% CI)	*P*		HR (95% CI)	*P*
Menopause status: menopause	*2.860 (1.057–7.736)*	*0.038*	Menopause status: menopause	2.609 (0.848–8.033)	0.095
Tumor PD-L1 expression: positive	*3.201 (1.273–8.050)*	*0.013*	Tumor PD-L1 expression: positive	2.413 (0.905–6.437)	0.078
CD3^+^ cell density: high	1.132 (0.475–2.701)	0.779	CD8^+^ cell density: low	2.003 (0.776–5.171)	0.151
CD8^+^ cell density: low	1.774 (0.709–4.438)	0.220			
FOXP3^+^ cell density: high	0.964 (0.405–2.294)	0.934			
PD1^+^ cell density: high	0.856 (0.363–2.021)	0.723			
Primary tumor size: ≥5 cm	1.956 (0.791–4.840)	0.147			
Lymph node invasion: positive	1.735 (0.385–7.811)	0.473			
Vascular invasion: positive	1.393 (0.576–3.365)	0.462			
Neural invasion: invasion	1.271 (0.535–3.016)	0.587			
Ovarian involvement: bilateral	1.926 (0.699–5.305)	0.205			

Tumor PD-L1 expression, CD3^+^ cell density, CD8^+^ cell density, FOXP3^+^ cell density, and PD1^+^ cell density are evaluated in KTs, and variables showing *P* values less than 0.05 are presented in italic.

**Table 2:** Univariate and multivariate analyses for prognostic factors affecting OS in CRC.

Risk factors	Univariate analysis		Risk factors	Multivariate analysis	
	HR (95% CI)	*P*		HR (95% CI)	*P*
Menopause status: menopause	2.437 (0.879–6.756)	0.087	Menopause status: menopause	3.381 (1.062–10.759)	*0.039*
Ratio of signet cells: high	4.655 (1.366–15.863)	*0.014*	Ratio of signet cells: high	2.301 (0.613–8.641)	0.217
Tumor PD-L1 expression: negative	5.129 (1.567–16.791)	*0.007*	Tumor PD-L1 expression: negative	4.451 (1.062–18.658)	*0.041*
Ovarian involvement: bilateral	1.155 (0.429–3.109)	0.776	CD8^+^ cell density: low	1.816 (0.462–7.140)	0.393
CD3^+^ cell density: low	1.640 (0.606–4.436)	0.330			
CD8^+^ cell density: low	3.408 (0.964–12.044)	0.057			
FOXP3^+^ cell density: high	1.316 (0.490–3.531)	0.586			
PD-1^+^ cell density: high	0.819 (0.301–2.230)	0.696			
Primary tumor size: ≥5 cm	0.399 (0.134–1.181)	0.097			
Lymph node invasion: positive	1.167 (0.329–4.138)	0.811			
Vascular invasion: positive	0.406 (0.127–1.295)	0.128			
Neural invasion: positive	3.926 (0.507–30.435)	0.191			

Tumor PD-L1 expression, CD3^+^ cell density, CD8^+^ cell density, FOXP3^+^ cell density, and PD1^+^ cell density are evaluated in KTs, and variables showing *P* values less than 0.05 are presented in italic.

## Data Availability

The data used to support the findings of this study are available from the corresponding author upon request.

## Supplementary Materials

Supplementary Materials Supplementary Table S1: correlation between the PD-L1 expression on the tumor or stroma and clinicopathological characteristics in KTs from GCs. Supplementary Table S2: correlation between the PD-L1 expression on the tumor or stroma and clinicopathological characteristics in KTs from CRCs. Supplementary Table S3: relation between the PD-L1 expression and T cell densities in KTs from GCs. Supplementary Table S4: relation between the PD-L1 expression and T cell densities in KTs from CRCs.
