# Supplementary material for: PD-L1 Expression Predicts a Distinct Prognosis in Krukenberg Tumor with Corresponding Origins
Source: J Immunol Res. 2018 May 8;2018:9485285. doi: 10.1155/2018/9485285 (PMC5964418; doi:10.1155/2018/9485285)
Supplement: Supplementary Materials — Supplementary Table S1: correlation between the PD-L1 expression on the tumor or stroma and clinicopathological characteristics in KTs from GCs. Supplementary Table S2: correlation between the PD-L1 expression on the tumor or stroma and clinicopathological characteristics in KTs from CRCs. Supplementary Table S3: relation between the PD-L1 expression and T cell densities in KTs from GCs. Supplementary Table S4: relation between the PD-L1 expression and T cell densities in KTs from CRCs. [file 9485285.f1.docx]

Supplementary Table S1: Correlation between PD-L1 expression on tumor or stoma and clinicopathological characteristics in KTs from GCs

|  |  | **PD-L1 expression** | | | | |  |
| --- | --- | --- | --- | --- | --- | --- | --- |
|  | **n** | **Tumor^pos^** | **Tumor^neg^** | ***P*** | **Stroma^pos^** | **Stroma^neg^** | ***P*** |
| **Total number** | 35 | 9 (25.7%) | 26 (74.3%) |  | 4 (11.4%) | 31 (88.6%) |  |
| **Age, years old** | 41 (22-62) | 40 (24-62) | 42 (22-58) |  | 47 (34-53) | 39 (22-62) |  |
| ≤40 | 17 (48.6%) | 3 (8.6%) | 14 (40.0%) | 0.443 | 1 (2.9%) | 16 (45.7%) | 0.603 |
| >40 | 18 (51.4%) | 6 (17.1%) | 12 (34.3%) |  | 3 (8.6%) | 15 (42.9%) |  |
| **Histological pathology** | | |  |  |  |  |  |
| Mix | 5 (14.3%) | 2 (5.7%) | 3 (8.6%) | 0.586 | 1 (2.9%) | 4 (11.4%) | 0.477 |
| Diffuse | 30 (85.7%) | 7 (20.0%) | 23 (65.7%) |  | 3 (8.6%) | 27 (77.1%) |  |
| **Menopause status** | |  |  |  |  |  |  |
| Premenopausal | 29 (82.9%) | 5 (14.3%) | 24 (68.6%) | **0.027** | 3 (8.6%) | 26 (74.3%) | 0.546 |
| Postmenopausal | 6 (17.1%) | 4 (11.4%) | 2 (5.7%) |  | 1 (2.9%) | 5 (14.3%) |  |
| **Ovarian involvement** | | |  |  |  |  |  |
| Bilateral | 21 (60.0%) | 6 (17.1%) | 15 (42.9%) | 0.712 | 3 (8.6%) | 18 (51.4%) | 0.635 |
| Unilateral | 14 (40.0%) | 3 (8.6%) | 11 (31.4%) |  | 1 (2.9%) | 13 (37.1%) |  |
| **Chronology** | |  |  |  |  |  |  |
| Synchronous | 29 (82.9%) | 9 (25.7%) | 20 (57.1%) | 0.304 | 4 (11.4%) | 25 (71.4%) | 1.000 |
| Metachronous | 6 (17.1%) | 0 (5.7%) | 6 (17.1%) |  | 0 (0.0%) | 6 (17.1%) |  |
| **Primary tumor location** | | |  |  |  |  |  |
| GEJ | 4 (11.4%) | 1 (2.9%) | 3 (8.6%) | 1.000 | 1 (2.9%) | 3 (8.6%) | 0.399 |
| Fundus/body | 31 (88.6%) | 8 (22.9%) | 23 (65.7%) |  | 3 (8.6%) | 28 (80.0%) |  |
| **Lymph node invasion** | | |  |  |  |  |  |
| Yes | 33 (94.3%) | 9 (25.7%) | 24 (68.6%) | 1.000 | 3 (8.6%) | 30 (85.7%) | 0.218 |
| No | 2 (5.7%) | 0 (0.0%) | 2 (5.7%) |  | 1 (2.9%) | 1 (2.9%) |  |
| **Vascular invasion** | |  |  |  |  |  |  |
| Yes | 14 (40%) | 6 (17.1%) | 8 (22.9%) | 0.112 | 3 (8.6%) | 11 (31.4%) | 0.279 |
| No | 21 (60%) | 3 (8.6%) | 18 (51.4%) |  | 1 (2.9%) | 20 (57.1%) |  |
| **Neural invasion** | |  |  |  |  |  |  |
| Yes | 11 (31.4%) | 3 (8.6%) | 8 (22.9%) | 0.144 | 3 (8.6%) | 8 (22.9%) | 0.082 |
| No | 24 (68.6%) | 6 (17.1%) | 18 (51.4%) |  | 1 (2.9%) | 23 (65.7%) |  |

*P* value was calculated by *χ^2^* test or Fisher’s exact test.

Supplementary Table S2: Correlation between PD-L1 expression on tumor or stoma and clinicopathological characteristics in KTs from CRCs

|  |  | **PD-L1 expression** | | | | |  |
| --- | --- | --- | --- | --- | --- | --- | --- |
|  | **n** | **Tumor^pos^** | **Tumor^neg^** | ***P*** | **Stroma^pos^** | **Stroma^neg^** | ***P*** |
| **Total number** | 30 | 20 (66.7%) | 10 (33.3%) |  | 8 (26.7%) | 22 (73.3%) |  |
| **Age (years, range)** | 50 (30-79) | 52 (30-73) | 45 (30-79) |  | 51 (42-61) | 50 (30-79) |  |
| **Primary site** |  |  |  | 1.000 |  |  | 0.351 |
| Colon | 25 (83.3%) | 16 (53.3%) | 9 (30.0%) |  | 6 (20.0%) | 19 (63.3%) |  |
| Rectum | 3 (10.0%) | 3 (10.0%) | 0 (0.0%) |  | 1 (3.3%) | 2 (6.7%) |  |
| Small intestine | 1 (3.3%) | 1 (3.3%) | 0 (0.0%) |  | 1 (3.3%) | 0 (0.0%) |  |
| Appendix | 1 (3.3%) | 0 (0.0%) | 1 (3.3%) |  | 0 (0.0%) | 1 (3.3%) |  |
| **Menopause status** |  |  |  | 0.442 |  |  | 0.689 |
| Premenopausal | 14 (46.7%) | 8 (26.7%) | 6 (20.0%) |  | 3 (10.0%) | 11 (36.7%) |  |
| Postmenopausal | 16 (53.3%) | 12 (40.0%) | 4 (13.3%) |  | 5 (16.7%) | 11 (36.7%) |  |
| **Ovarian involvement** | |  |  | 0.700 |  |  | 0.682 |
| Bilateral | 15 (50.0%) | 9 (30.0%) | 6 (20.0%) |  | 3 (10.0%) | 12 (40.0%) |  |
| Unilateral | 15 (50.0%) | 11 (36.7%) | 4 (13.3%) |  | 5 (16.7%) | 10 (33.3%) |  |
| **Chronology** |  |  |  | 1.000 |  |  | 1.000 |
| Synchronous | 28 (93.3%) | 19 (63.3%) | 9 (30.0%) |  | 8 (26.7%) | 20 (66.7%) |  |
| Metachronous | 2 (6.7%) | 1 (3.3%) | 1 (3.3%) |  | 0 (0.0%) | 2 (6.7%) |  |
| **Extent of signet ring cells** | |  |  | **0.009** |  |  | 0.143 |
| 1-20% | 24 (80.0%) | 19 (63.3%) | 5 (16.7%) |  | 8 (26.7%) | 15 (50.0%) |  |
| >20% | 6 (20.0%) | 1 (3.3%) | 5 (16.7%) |  | 0 (0.0%) | 7 (23.3%) |  |
| **Primary tumor diameter (cm)** | |  |  | 1.000 |  |  | 0.256 |
| ≤5 | 24 (80.0%) | 15 (50.0%) | 9 (30.0%) |  | 8 (26.7%) | 16 (53.3%) |  |
| 5-10 | 5 (16.7%) | 4 (13.3%) | 1 (3.3%) |  | 0 (0.0%) | 5 (16.7%) |  |
| ≥10 | 1 (3.3%) | 1 (3.3%) | 0 (0.0%) |  | 0 (0.0%) | 1 (3.3%) |  |
| **Tumor differentiation** | |  |  | 1.000 |  |  | 0.417 |
| Moderate | 16 (53.3%) | 11 (36.7%) | 5 (16.7%) |  | 3 (10.0%) | 13 (43.3%) |  |
| Poor | 14 (46.7%) | 9 (30.0%) | 5 (16.7%) |  | 5 (16.7%) | 9 (30.0%) |  |
| **Lymph node invasion** | |  |  | 1.000 |  |  | 0.643 |
| Yes | 22 (73.3%) | 15 (50.0%) | 7 (23.3%) |  | 5 (16.7%) | 17 (56.7%) |  |
| No | 8 (26.7%) | 5 (16.7%) | 3 (10.0%) |  | 3 (10.0%) | 5 (16.7%) |  |
| **Vascular invasion** |  |  |  | 0.694 |  |  | 0.678 |
| Yes | 12 (40.0%) | 9 (30.0%) | 3 (10.0%) |  | 4 (13.3%) | 8 (26.7%) |  |
| No | 18 (60.0%) | 11 (36.7%) | 7 (23.3%) |  | 4 (13.3%) | 14 (46.7%) |  |
| **Neural invasion** |  |  |  | 1.000 |  |  | 1.000 |
| Yes | 4 (13.3%) | 3 (10.0%) | 1 (3.3%) |  | 1 (3.3%) | 3 (10.0%) |  |
| No | 26 (86.7%) | 17 (56.7%) | 9 (30.0%) |  | 7 (23.3%) | 19 (63.3%) |  |

Supplementary Table S3: Relation between PD-L1 expression and T cell densities in KTs from GCs

|  |  | Tumor PD-L1 expression | |  |  | Stromal PD-L1 expression | |  |
| --- | --- | --- | --- | --- | --- | --- | --- | --- |
|  | Total number | Positive  9 (25.7%) | Negative  26 (74.3%) | *P* | Total number | Positive  4 (11.4%) | Negative  31 (88.6%) | *P* |
| CD3^+^ cell density | |  |  | 1.000 | |  |  | 0.338 |
| Low | 17 (48.6%) | 4 (11.4%) | 13 (37.1%) |  | 17 (48.6%) | 3 (8.6%) | 14 (40.0%) |  |
| High | 18 (51.4%) | 5 (14.3%) | 13 (37.1%) |  | 18 (51.4%) | 1 (2.9%) | 17 (48.6%) |  |
| CD8^+^ cell density | |  |  | 1.000 | |  |  | 0.603 |
| Low | 18 (51.4%) | 5 (8.6%) | 13 (42.9%) |  | 18 (51.4%) | 3 (8.6%) | 15 (42.9%) |  |
| High | 17 (48.6%) | 4 (17.1%) | 13 (31.4%) |  | 17 (48.6%) | 1 (2.9%) | 16 (45.7%) |  |
| FOXP3^+^ cell density | |  |  | 0.443 | |  |  | 0.603 |
| Low | 18 (51.4%) | 6 (17.1%) | 12 (34.3%) |  | 18 (51.4%) | 3 (8.6%) | 15 (42.9%) |  |
| High | 17 (48.6%) | 3 (8.6%) | 14 (40.0%) |  | 17 (48.6%) | 1 (2.9%) | 16 (45.7%) |  |
| PD1^+^ cell density | |  |  | 0.711 | |  |  | 1.000 |
| Low | 18 (51.4%) | 4 (11.4%) | 14 (40.0%) |  | 18 (51.4%) | 2 (5.7%) | 16 (45.7%) |  |
| High | 17 (48.6%) | 5 (14.3%) | 12 (34.3%) |  | 17 (48.6%) | 2 (5.7%) | 15 (42.9%) |  |

Supplementary Table S4: Relation between PD-L1 expression and T cell densities in KTs from CRCs

|  |  | **Tumor PD-L1 expression** | |  |  | **Stromal PD-L1 expression** | |  |
| --- | --- | --- | --- | --- | --- | --- | --- | --- |
|  | **Total number** | **Positive**  **20 (66.7%)** | **Negative**  **10 (33.3%)** | ***P*** | **Total number** | **Positive**  **8 (26.7%)** | **Negative**  **22 (73.3%)** | *P* |
| **CD3^+^ cell density** | |  |  | 0.700 |  |  |  | 0.682 |
| Low | 15 (50.0%) | 9 (30.0%) | 6 (20.0%) |  | 15 (50.0%) | 5 (16.7%) | 10 (33.3%) |  |
| High | 15 (50.0%) | 11 (36.7%) | 4 (13.3%) |  | 15 (50.0%) | 3 (10.0%) | 12 (40.0%) |  |
| **CD8^+^ cell density** | |  |  | 0.702 |  |  |  | 1.000 |
| Low | 19 (63.3%) | 12 (40.0%) | 7 (23.3%) |  | 14 (46.7%) | 4 (13.3%) | 10 (33.3%) |  |
| High | 11 (36.7%) | 8 (26.7%) | 3 (10.0%) |  | 16 (53.3%) | 4 (13.3%) | 12 (40.0%) |  |
| **FOXP3^+^ cell density** | |  |  | 0.705 |  |  |  | 1.000 |
| Low | 17 (56.7%) | 12 (40.0%) | 5 (16.7%) |  | 17 (56.7%) | 5 (16.7%) | 12 (40.0%) |  |
| High | 13 (43.3%) | 8 (26.7%) | 5 (16.7%) |  | 13 (43.3%) | 3 (10.0%) | 10 (33.3%) |  |
| **PD1^+^ cell density** | |  |  | 1.000 |  |  |  | 1.000 |
| Low | 19 (63.3%) | 13 (43.3%) | 6 (20.0%) |  | 19 (63.3%) | 5 (16.7%) | 14 (46.7%) |  |
| High | 11 (36.7%) | 7 (23.3%) | 4 (13.3%) |  | 11 (36.7%) | 3 (10.0%) | 8 (26.7%) |  |
